# CLIC5A binds to and stabilizes the open and active conformation of ezrin

**DOI:** 10.1016/j.jbc.2025.110646

**Published:** 2025-08-28

**Authors:** Md. Mizanur Rahman, Jong S. Kim, Laiji Li, M. Rafid Feisal, Kevin Y.L. Mak, Mahtab Tavasoli, Zhixiang Wang, Barbara J. Ballermann, Peter M. Hwang

**Affiliations:** 1 Department of Medicine, University of Alberta, Edmonton, Canada; 2 Department of Genetics, University of Alberta, Edmonton, Canada; 3 Department of Physiology, University of Alberta, Edmonton, Canada; 4 Department of Biochemistry, University of Alberta, Edmonton, Canada

**Keywords:** actin cytoskeleton, ezrin, ERM proteins, chloride intracellular channel, Rac1-GTP

## Abstract

Ezrin, radixin, and moesin (ERM) proteins regulate assembly of actin-based structures, link membrane-spanning proteins to cortical actin, and are part of cell signaling hubs. The chloride intracellular channel (CLIC) 5A protein is very abundant in radixin-dependent inner ear hair cell stereocilia and in ezrin-dependent kidney glomerular podocyte foot processes and is essential for the structural integrity of these actin-based cellular projections. The functional relationship between ERM proteins and CLIC5A is incompletely understood and whether CLIC5A functions as a chloride channel is controversial. We determined whether CLIC5A is membrane-spanning protein and sought direct CLIC5A binding partners. While CLIC5A localized predominantly to the dorsal plasma membrane domain, we found CLIC5A to be a soluble, intracellular protein, without characteristics expected of a membrane-spanning channel. In the yeast two-hybrid assay, CLIC5A interacted directly with the C-terminal domains of ERM with a hierarchy of ezrin > radixin = moesin. The last 16 amino acids of ezrin were essential but not sufficient for CLIC5A binding, and phosphorylation of ezrin at T567 enhanced the interaction. The affinity of purified CLIC5A for a phosphomimetic ezrin_482-586_ (T567E) C-terminal fragment was in the 30 μM range. Silencing of ERM dislodged CLIC5A from the peripheral location, and the CLIC5A–ezrin interaction augmented Rho guanine nucleotide dissociation inhibitor sequestration by ezrin and Rac1 activity. Thus, CLIC5A functions as a direct binding partner of ezrin, stabilizing its open/active conformation and resulting in localized small GTPase activation.

Chloride intracellular channel (CLIC) proteins (1, 2) are characterized by a conserved ∼240-aa “CLIC” two-domain structure most closely resembling ω-type glutathione S-transferases (GSTs). CLICs exist in vertebrates, invertebrates (3, 4), plants (5), and bacteria (6). There are 6 mammalian *CLIC* genes (*CLIC1–CLIC6*). Among these, the *CLIC5* gene produces two known isoforms through alternative usage of distinct exons 1A and 1B generating CLIC5A (7) (251 aa) and CLIC5B (8, 9) (410 aa, aka p64), respectively.

The membrane-spanning nature of CLIC proteins remains controversial (10). Recombinant CLICs can partition into artificial lipid bilayers and facilitate nonselective ion flux (2, 11, 12). However, even though transiently expressed CLIC5A localizes near the apical plasma membrane in cultured cells, its presence in cells does not alter anion efflux (11). Additionally, CLIC proteins are produced without signal peptide, usually required for insertion into the plasma membrane, and crystal structures of the closely related CLIC1 (13), CLIC2 (14), and CLIC4 (15) family members predict that they are soluble proteins that do not possess even a single predicted transmembrane helix. Hence, CLICs do not resemble classic ion channels. To explain their effect on ion conductance, CLICs were postulated to undergo a redox-sensitive unfolding transition producing oligomers that can spontaneously insert into lipid bilayers. CLICs also associate with intracellular organelles and cytoskeletal proteins (7, 16, 17), but their molecular mechanisms of action remain poorly understood (2).

CLIC5A was first identified as a component of the actin cytoskeleton in human placental microvilli, localizing to the apical domain of trophoblasts (7). It is an essential component of inner ear sensory stereocilia (18). The actin-based stereocilia project from the apical surface of the hair cells serving to sense sound in the cochlea and head position in the vestibular system. The CLIC5A protein is also highly enriched in kidney glomeruli (17, 19), where it localizes to the apical domain of podocyte foot processes *in vivo* (17). Podocyte foot processes are actin-based projections that wrap around the exterior of glomerular capillaries, providing structural integrity and opposing protein filtration.

Two human families with loss-of-function *CLIC5* mutations have been reported (20, 21, 22), with a total of five affected individuals who all suffer from nonsyndromic sensorineural hearing loss starting early in life and progressing to deafness by the second decade (21, 22). Two individuals also had vestibular abnormalities, and one had kidney dysfunction. In “jitterbug” (*jbg/jbg*) mice, a partial deletion of *CLIC5* exon 5 causes CLIC5A and CLIC5B proteins to be absent and results in progressive degeneration of inner ear hair cell sensory stereocilia with vestibular dysfunction at birth and eventual deafness (18, 23). In the kidney of CLIC5-deficient mice, the number of glomerular podocyte foot processes is reduced, and they are shorter and broader than those in WT mice (17, 19). CLIC5 deletion in mice strongly enhances their susceptibility to hypertension-induced damage to glomerular capillaries (24).

Berryman *et al.* (7, 11) first identified CLIC5A in a cytoskeletal protein complex pulled from placental microvilli by a short 36-aa C-terminal ezrin fragment. We observed that when CLIC5A is expressed in COS-7 cells, ezrin, radixin, moesin (ERM) protein phosphorylation increases along with enhanced actin polymerization, the formation of surface ruffles and dorsal plasma membrane phosphatidylinositol 4,5 bisphosphate [PI(4,5)P_2_] clusters (25). Deletion of CLIC5 *in vivo* results in a time-dependent decrease of ezrin protein abundance in glomeruli (17), decreased podocyte ERM phosphorylation (24, 26) and dissociation of the glomerular ezrin/NHERF2/podocalyxin complex (25). CLIC5A expression also enhances Rac1 activation and phosphorylation of its downstream effector Pak1/3 (24). This is consistent with another study in which CLIC1 and CLIC4 proteins contributed to Rac1 activation in cultured endothelial cells (27). Given that Rac1-GTP activates PI4P5 kinases, which produce PI(4,5)P_2_ required for ERM protein activation at the cell membrane, we have suggested a model for CLIC5A action in which CLIC5A-dependent Rac1 activation leads to PI4P5 kinase–dependent PI(4,5)P_2_ generation and consequent ERM activation and phosphorylation (24).

This study first sought to determine whether CLIC5A can span the plasma membrane as would be expected for a plasma membrane chloride channel, and the findings indicate that CLIC5A is exclusively intracellular. We found that CLIC5A interacts directly with ERM C-terminal tails. The extreme C-terminal 16 aa of ezrin are required for CLIC5A binding, ezrin phosphorylation at T567 promotes the ezrin/CLIC5A interaction but the autoinhibited conformation of soluble, full-length ezrin does not support CLIC5A binding. Both CLIC5A and ezrin coprecipitated with Rac1-GTP, indicating that they are part of the same protein complex. While the interaction of CLIC5A with Rac1-GTP was indirect, Rho guanine nucleotide dissociation inhibitor (Rho-GDI) sequestration by ezrin was enhanced by CLIC5A. The findings lead us to conclude that CLIC5A functions as a direct interaction partner for ezrin, stabilizing its active conformation, leading to Rac1 activation.

## Results

### Ectopically expressed CLIC5A is not found at the COS-7 cell surface

**Figure 1:**
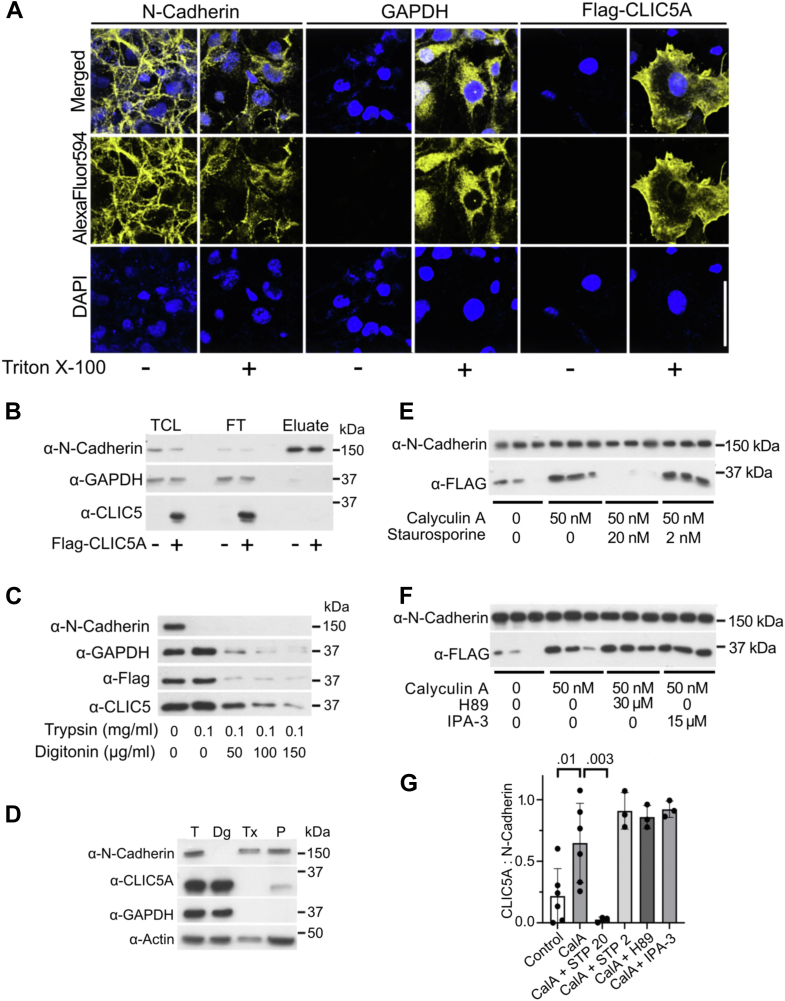
**Expressed FLAG-CLIC5A is a soluble intracellular protein.***A*, anti(α)-N-cadherin, α-GAPDH, and α-FLAG immunoreactivity (yellow) in COS-7 cells with transiently expressed FLAG-CLIC5A ± Triton X-100 permeabilization. Nuclei were visualized with DAPI (*blue*). The scale bar represents 50 μm. *B*, surface protein biotinylation in nonpermeabilized COS-7 cells transfected with FLAG-CLIC5A or vector cDNA. Biotinylated proteins were captured on an avidin affinity column. Total cell lysate (TCL), proteins in the flow-through (FT), and proteins eluted from the avidin column (eluate) were subjected to WB analysis for endogenous N-cadherin and GAPDH and transiently expressed Flag-CLIC5A. *C*, WB of lysates from COS-7 cells transfected with *FLAG-CLIC5A* cDNA and treated with trypsin (30 min, on ice) without or with digitonin permeabilization. Blots show endogenous N-cadherin, GAPDH, and transiently expressed Flag-CLIC5A. *D*, differential detergent fractionation followed by WB analysis of COS-7 cells transiently expressing untagged CLIC5A. Digitonin (D) releases soluble proteins, Triton X-100 (T) releases lipid-bound proteins and the pellet (P) represents insoluble proteins in the cytoskeleton and some subcellular organelles. Endogenous N-cadherin serves as control for integral membrane proteins and endogenous GAPDH as control for soluble proteins. CLIC5A is observed in the digitonin soluble fraction and in the pellet. *E*–*F*, WBs for endogenous N-cadherin and Flag-CLIC5A in Triton X-100 soluble fractions from COS-7 cells transfected with *FLAG-CLIC5A* cDNA and treated with vehicle or the Ser/Thr phosphatase inhibitor calyculin A (CalA, 50 nM) for 20 min prior to lysis. Cells were treated with vehicle or staurosporine (STP, 20 and 2 nM) or vehicle (*E*), H89 (30 μM) or IPA-3 (15 μM) (*F*) for 30 min prior to addition of CalA. Each lane represents a biologically independent experiment. *G*, quantification of Flag-CLIC5A in the Triton X-100 soluble fractions relative to N-cadherin. *A*-*D*, representative of three biologically distinct experiments; (*E*-*F*) n = 3 independent experiments, (*G*) mean ± SD, one-way ANOVA (F = 11.26; *p* < 0.0001; *post hoc* Dunnett’s multiple comparisons *p* values are shown). CLIC, chloride intracellular channel; DAPI, 4′,6-diamidino-2-phenylindole; IPA-3, 1,1′-Dithiodi-2-naphthtol; WB, Western blot.

To clarify whether the N terminus of CLIC5A extends through the plasma membrane to the cell surface, nonpermeabilized and permeabilized *FLAG-CLIC5A* complementary DNA (cDNA)-transfected COS-7 cells were subjected to immunofluorescence microscopy with anti-FLAG, anti-GAPDH, and anti-N-cadherin antibodies (Fig. 1*A*). While the membrane-spanning N-cadherin was readily detected in nonpermeabilized cells, FLAG-CLIC5A and GAPDH were only detected in permeabilized cells, indicating that the FLAG epitope at the N terminus of CLIC5A remains intracellular.

We next determined whether CLIC5A can be biotinylated in nonpermeabilized cells. Intact COS-7 cells transfected with vector or *FLAG-CLIC5A* cDNA were treated with a membrane-impermeant biotin derivative that labels only cell surface proteins with accessible lysine residues, followed by capture of biotinylated proteins on immobilized avidin. Whereas endogenous N-cadherin was captured by avidin after surface biotinylation, CLIC5A was observed only in the flow-through and was not captured by avidin (Fig. 1*B*) like endogenous cytosolic GAPDH. Hence, no accessible lysine residues in CLIC5A are present at the cell surface.

We also determined whether CLIC5A is subject to proteolytic degradation in intact cells. FLAG-CLIC5A expressing COS-7 cells were left intact or were permeabilized with increasing concentrations of digitonin and treated with trypsin. In the absence of digitonin, N-cadherin was readily degraded by trypsin, whereas FLAG-CLIC5A and GAPDH remained intact (Fig. 1*C*). Both FLAG-CLIC5A and GAPDH were degraded by trypsin in cells that were digitonin-permeabilized. Hence, CLIC5A is not available at the cell surface for cleavage by extracellular trypsin.

### Phosphorylation-dependent CLIC5A association with the Triton X-100 soluble fraction

We next performed cell fractionation experiments to determine whether CLIC5A can associate with the Triton X-100 soluble “membrane” fraction in COS-7 cells (Fig. 1, *D*–*G*). GAPDH was observed exclusively in the digitonin-soluble fraction, whereas N-cadherin was observed in the Triton X-100 soluble fraction and in the digitonin/Triton X-100–resistant pellet. Nearly all the CLIC5A transiently expressed in COS-7 cells was recovered in the digitonin-soluble fraction, with an extremely small portion in the digitonin/Triton X-100 insoluble fraction (Fig. 1*D*). Like CLIC5A, we observed that endogenous CLIC4 and CLIC1 similarly partitioned into the digitonin soluble fraction of COS-7 cells (Fig. S2) (28).

To determine whether phosphorylation alters the association of CLIC5A with the Triton X-100 soluble fraction in COS-7 cells, the abundance of transiently expressed FLAG-CLIC5A relative to endogenous N-cadherin in the Triton X-100 fraction was determined (Fig. 1*E*). Since the very small amounts of CLIC5A were initially not detected in the Triton X-100 fraction, 5-fold more lysate was loaded in these experiments than that in those shown in Figure 1*D*, and only the Triton X-100 fractions were compared. The abundance of FLAG-CLIC5A in the Triton X-100 soluble fraction increased in cells treated with the Ser/Thr phosphatase inhibitor calyculin A for 20 min. In cells pretreated with 20 nM staurosporine, a relatively nonspecific kinase inhibitor, and then subjected to calyculin A, CLIC5A was undetectable in the Triton X-100 soluble fraction. By contrast, pretreatment with 2 nM staurosporine, more specific for PKC, the PKA inhibitor H89 or the PAK inhibitor 1,1′-Dithiodi-2-naphthtol did not reduce the calyculin-A–stimulated association of CLIC5A with the Triton X-100 soluble fraction (Fig. 1*, E* –*G*). Thus, although transfected CLIC5A is predominantly soluble, a small increase in membrane association was brought about when pSer/pThr dephosphorylation was blocked.

### CLIC5A interacts directly with ERM proteins

**Figure 2:**
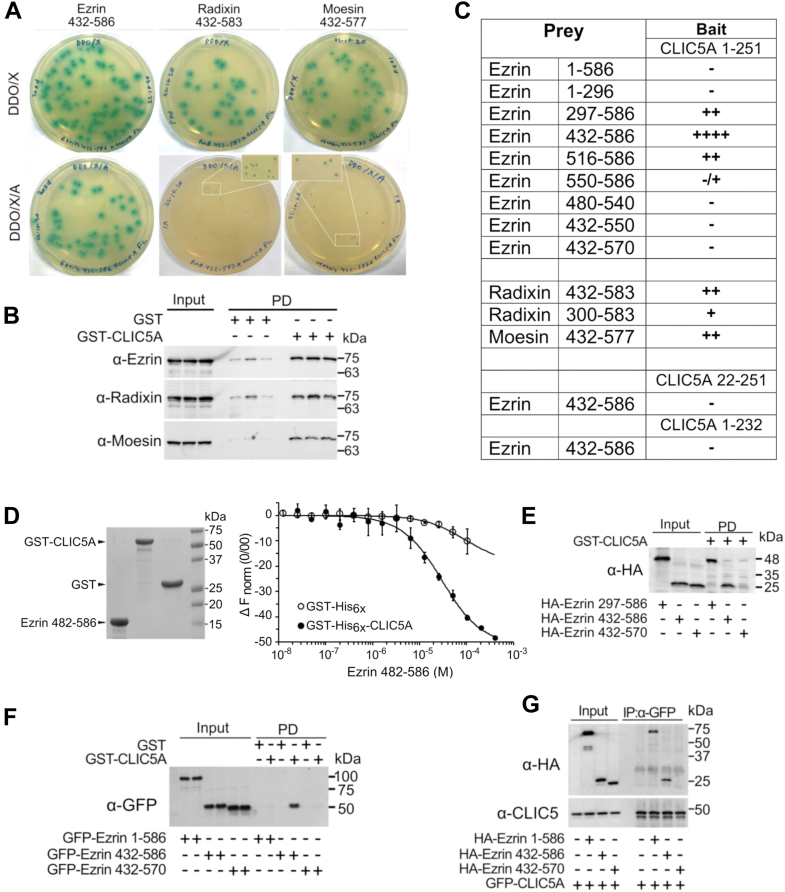
**CLIC5A interacts directly with ezrin, radixin, and moesin.***A*. yeast two-hybrid (Y2H) assay. Plates contain yeast colonies expressing untagged CLIC5A from the “bait” vector and ezrin_432-586_, radixin_432-583_, or moesin_432-577_ from the “prey” vector. Colony growth on double-deficient medium (DDO, lacking leucine and tryptophan) indicates both vectors are present. *Blue* colonies indicate induced α-galactosidase activity in the presence of X-α-gal (DDO/X) due to direct interactions between bait and prey proteins. Growth on plates containing the antibiotic aureobasidin A (DDO/X/A) indicate a direct interaction between bait and prey proteins resulting in Aureobasidin A resistance. The inset for DDO/X/A plates containing CLIC5A/radixin_432-583_ or CLIC5A/moesin_432-577_ represents a 2× digital magnification (representative of 3 biologically separate experiments). *B*, WB with anti(α)-ezrin, α-radixin and α-moesin antibodies of total cell lysates (input) and GST or GST-CLIC5A pulldowns (PD) from untransfected COS-7 cells. Each of the three lanes for input, GST pulldown, and GST-CLIC5A pulldown are from three biologically distinct experiments. *C*, semiquantitative Y2H mapping of interactions between distinct ERM and CLIC5A constructs. Input and GST-CLIC5A pulldown (PD) is shown (representative of three independent experiments). *D*, microscale thermophoresis (MST) *left panel:* total protein stain of recombinant, purified GST-His6x, GST-His6x-CLIC5A, and untagged ezrin_432-586_ proteins. *Right panel:* normalized thermophoresis-induced change in fluorescence for fluorescently labeled GST-His6x or GST-His6x-CLIC5A in the presence of increasing concentrations of purified ezrin_482-586_ (H549N/T567E). The calculated affinity (Kd) between GST-His6x-CLIC5A and ezrin_432-586_ (H549N/T567E) was 29 ± 12 μM (mean ± SD, n = 3 biological replicates). *E*, WB of HA-ezrin_297-586_, HA-ezrin_432-586_, and HA-ezrin_432-570_ produced by *in vitro* transcription/translation before (input) and after pulldown (PD) by immobilized GST-CLIC5A. *F*, α-GFP WB of transiently expressed full-length GFP-ezrin_1-586_, GFP-ezrin_432-586_, and GFP-ezrin_432-570_ in COS-7 cells and pulled from the cell lysates by immobilized GST or GST-CLIC5A (PD) (representative of three biologically independent experiments). *G*, coimmunoprecipitation of HA-ezrin_1-586_, HA-ezrin_432-586_, and HA-ezrin_432-570_ coexpressed with GFP-CLIC5A in COS-7 cells (representative of three biological replicates). CLIC, chloride intracellular channel; DDO, double dropout; ERM, ezrin, radixin, and moesin; GST, glutathione S-transferase; WB, Western blot.

A yeast two-hybrid (Y2H) screen of a mouse kidney protein domain library (Hybrigenics, Evry) identified the radixin C-terminal domain (aa 330–583) as one potential direct binding partner of CLIC5A. In subsequent Y2H assays, coexpression of CLIC5A as bait together with the C termini of human ERM (ezrin_432-586_, radixin_432-583_, moesin_432-577_) as prey induced expression of the MEL1/α-galactosidase reporter resulting in blue colonies in the presence of X-alpha-gal (Fig. 2*A*), confirming direct interactions between CLIC5A and C-terminal fragments of ERM. Consistent with the Y2H findings, purified, immobilized GST-CLIC5A pulled endogenous ERM from HeLa cell lysates (Fig. 2*B*). However, while growth on the antibiotic aureobasidin A, which requires induction of the reporter AUR1-C through a direct bait/prey interaction, was also observed when the CLIC5A bait was coexpressed with ERM C-terminal fragments as prey, the number and size of blue colonies was consistently (n = 3 distinct experiments) greater for the CLIC5A/ezrin_432-586_ combination than the combination of CLIC5A/radixin_432-583_ or CLIC5A/moesin_432-577_, the latter producing fewer and smaller blue colonies (Fig. 2*A*). This suggested that the affinity of CLIC5A for ezrin_432-586_ is greater than its affinity for radixin_432-583_ or moesin_432-577_. The interaction between CLIC5A and ezrin was therefore studied in more detail. Mapping of the CLIC5A/ezrin interaction by Y2H assay (Fig. 2*C*) revealed no interaction of CLIC5A with full-length ezrin (ezrin_1-586_), the N-terminal FERM domain of ezrin (ezrin_1-296_), or the ezrin C terminus in which the last 16 amino acids were deleted (ezrin_432-570_), and the interaction of CLIC5A with ezrin_550-586_ was weak. N- and C-terminal deletion mutants of CLIC5A (CLIC5A_22-251_ and CLIC5A_1-232_) also failed to interact with ezrin_432-586_ (Fig. 2*C*).

The affinity between the ezrin C terminus and CLIC5A was quantified by microscale thermophoresis (MST) (Fig. 2*D*). When purified, fluorescently labeled GST-His_6x_-CLIC5A was incubated with unlabeled, purified ezrin_482-586_ (left panel) in the absence of other cell constituents, the dissociation constant (K_d_) determined by MST averaged 29 ± 12 μM (mean ± SD, n = 3) (Fig. 2*D*, right panel). Ezrin_482-586_ did not alter the signal from fluorescently labeled control GST-His_6x_ sufficiently to allow a K_d_ determination (Fig. 2*D*, right panel), and no interaction between fluorescent His_6x_ peptide and ezrin_482-586_ was detected (not shown).

Consistent with the Y2H findings, purified, immobilized GST-CLIC5A effectively precipitated *in vitro* transcribed and translated ezrin_432-586_ and ezrin_297-586_, but not ezrin_432-570_ (Fig. 2*E*). When GFP-ezrin constructs were expressed in COS-7 cells, immobilized GST-CLIC5A pulled GFP-ezrin_432-586_, but not GFP-ezrin_1-586_ or GFP-ezrin_432-570_ (Fig. 2*F*) from the cell lysates, also consistent with the Y2H findings. Nonetheless, when coexpressed with GFP-CLIC5A in COS-7 cells, full-length HA-ezrin_1-586_, HA-ezrin_432-586_, but not ezrin HA ezrin_432-570,_ coimmunoprecipitated GFP-CLIC5A (Fig. 2*G*).

Thus, CLIC5A can interact directly with the ezrin C terminus, with an affinity in the 30 μM range. The terminal 16 amino acids of ezrin are required but not sufficient for CLIC5A binding. CLIC5A interactions with full-length ezrin_1-586_ were variable. GST-CLIC5A was able to pull endogenous ezrin (as well as moesin and radixin) from cell lysates (Fig. 2*B*), but full-length tag-less ezrin_1-586_ did not interact with CLIC5A in the Y2H assay (Fig. 2*C*) and expressed GFP-ezrin_1-586_ was not pulled from cell lysates by immobilized GST-CLIC5A. Even so, HA-ezrin_1-586_ coimmunoprecipitated with GFP-CLIC5A when both proteins were coexpressed in cells (Fig. 2*G*). The findings suggest that CLIC5A does not bind soluble ezrin in the autoinhibited state, known to result from a high-affinity interaction between ezrin N-terminal FERM and C-terminal domains (29). We therefore postulate that the C terminus of ezrin must be freed from autoinhibition, to allow CLIC5A binding.

### The peripheral CLIC5A localization is partly mediated by ERM proteins

**Figure 3:**
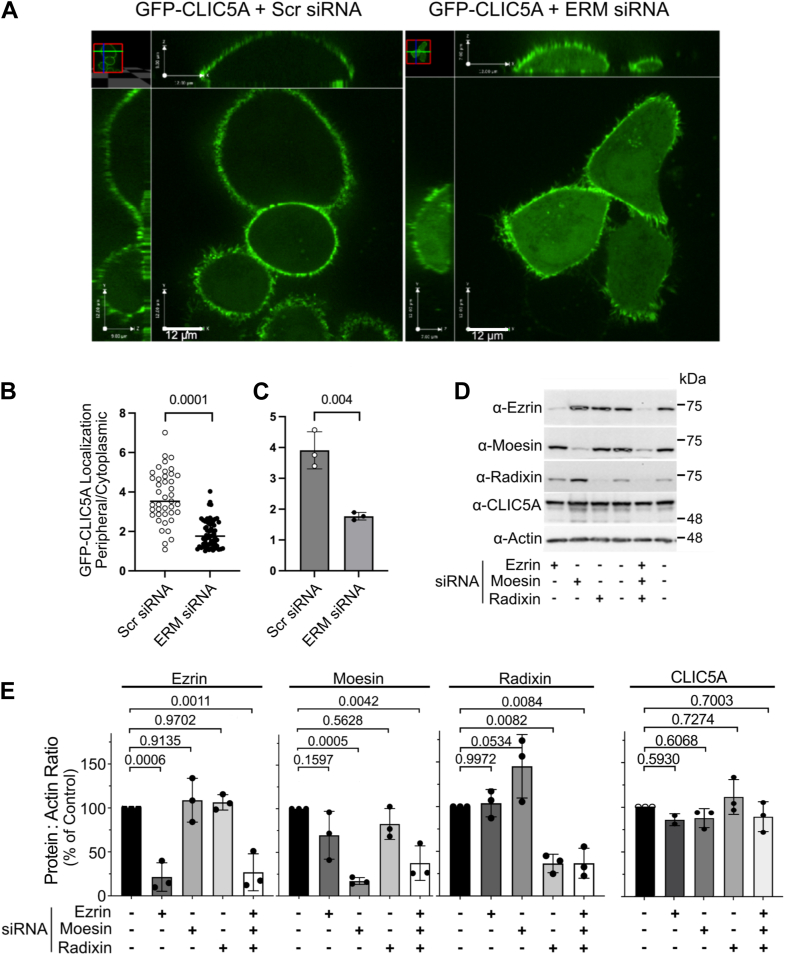
**ERM knockdown partially displaces CLIC5A from its peripheral location.***A*, localization of transiently expressed GFP-CLIC5A in living HeLa cells transfected with nonspecific siRNA (Scr siRNA) or triple ezrin, moesin, and radixin siRNA (ERM siRNA) (representative of three biologically distinct experiments). *B*, quantification of the peripheral: cytoplasmic GFP-CLIC5A ratio in living HeLa cells transfected with GFP-CLIC5A and with Scr siRNA or triple ERM siRNA from a single experiment (Student’s *t* test). *C*, mean peripheral: cytoplasmic GFP-CLIC5A in living HeLa cells transfected with GFP-CLIC5A and Scr siRNA or triple ERM siRNA (mean ± SD, n = 3 biologically independent experiments, Student’s *t* test). *D:* WB with anti-ezrin, anti-radixin, and anti-moesin antibodies showing individual and triple ezrin, radixin, and moesin siRNA-mediated knockdown of endogenous ezrin, radixin and moesin in HeLa cells. *E*: Quantification of ERM knockdown from three independent experiments. (One-way ANOVA for ezrin abundance: F = 21.17; *p* < 0.001, for moesin abundance: F = 11.50; *p* < 0.0009, for radixin abundance: F = 17.68; *p* < 0.0002, and for CLIC5A abundance: F = 1.564; *p* = 0.265. *p* values for *post hoc* Dunnett’s multiple comparisons are shown). CLIC, chloride intracellular channel.

We next determined whether CLIC5A might be recruited to the cell periphery by ERM proteins. In HeLa cells, CLIC5A localized predominantly to the dorsal cell periphery (Fig. 3, *A* and *B*), similar to previous observations in COS-7 cells (25). However, though ezrin was effectively silenced in HeLa cells, no consistent effect on the location of CLIC5A was observed (not shown). Since ERM proteins are all pulled down from cell lysates by GST-CLIC5A (Fig. 2*B*), and since they all interact with CLIC5A in the Y2H assay (Fig. 2, *A* and *C*), we determined whether triple siRNA-mediated knockdown of endogenous ERM changes the subcellular distribution of CLIC5A. Triple ERM silencing was 75 to 85% effective (Fig. 3*C*). A significantly larger portion of transiently expressed GFP-CLIC5A remained cytosolic in cells with triple ERM silencing compared to cells treated with nonspecific siRNA (Fig. 3, *A* and *B*), though a significant portion of GFP-CLIC5A remained at the cell periphery. Therefore, the peripheral localization of CLIC5A in HeLa cells depends, at least in part, on ERM protein expression. Incomplete elimination of peripheral GFP-CLIC5A by triple siRNA ERM knockdown could result from residual expression of ERM proteins, but other mechanisms not related to ERM proteins could also target CLIC5A to the cell periphery.

### Ezrin phosphorylation increases its interaction with CLIC5A

**Figure 4:**
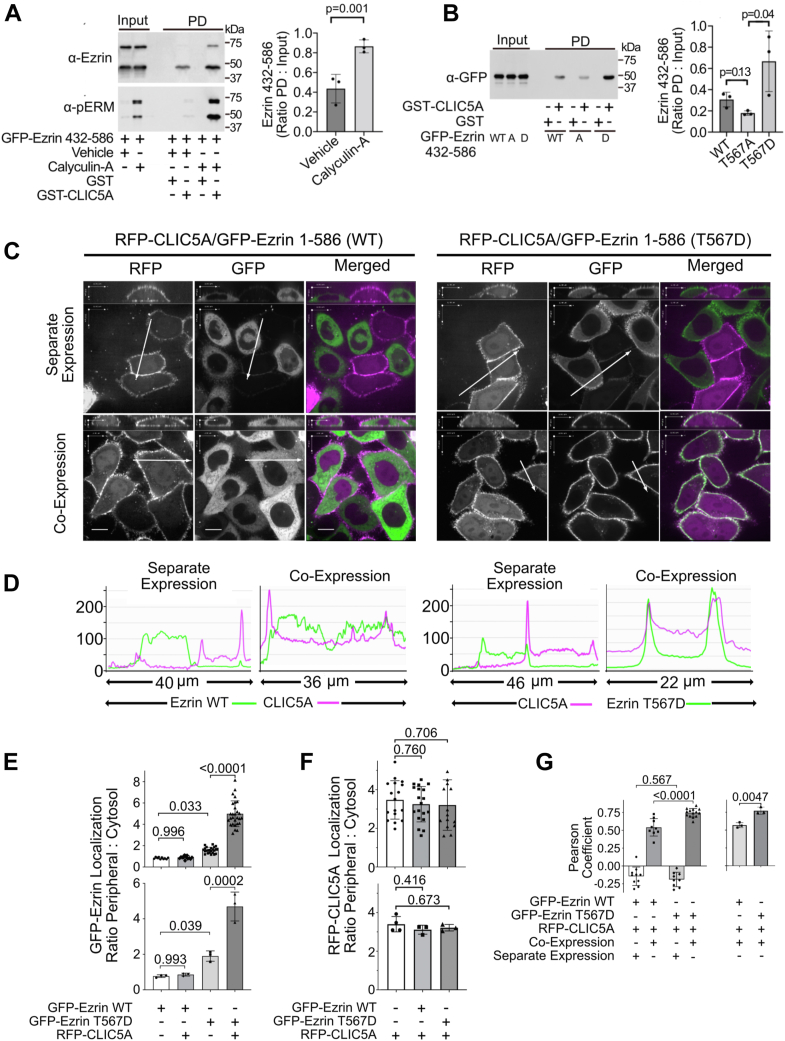
**Phosphorylation enhances the CLIC5A/ezrin interaction.***A*, *left panel:* WB for endogenous ezrin and endogenous phosphorylated ERM proteins (pERM) in COS-7 cell lysates and GST-CLIC5A pulldowns (PD) from cells treated with vehicle or calyculin A (50 nM) for 30 min prior to lysis. *Right panel:* densitometric quantification (mean ± SD, n = 3 independent experiments, Student’s *t* test). *B*, *right panel:* α-GFP WB of total cell lysate (input) and GST or GST-CLIC5A pulldown (PD) from lysates of COS-7 transiently expressing GFP-ezrin_432-586_ (WT), phosphorylation deficient GFP-ezrin_432-586_ (T567A; “A”) or phosphomimetic GFP-ezrin_432-586_ (T567D; “D”). *Right panel:* densitometric quantification of GST-CLIC5A pulldown of transiently expressed GFP-ezrin_432-586_ (WT, A or D mutants; one-way ANOVA F = 5.42; *p* = 0.04, *p* values for *post hoc* Dunnett’s multiple comparisons are shown). *C*, confocal fluorescence microscopy of living HeLa cells transfected with RFP-CLC5A (*magenta*) and/or full-length GFP-ezrin_1-586_ (*green*) without (WT) or with a phosphomimetic T567D mutation (the scale bar represents 12 μm). Cells were either transfected separately (*top row*) with RFP-CLIC5A or GFP-ezrin and then mixed 1:1 (control conditions) or they were cotransfected (*bottom row*) with RFP-CLIC5A and GFP-ezrin. The *white arrows* represent line plots analyzed for pixel intensity (representative of three biologically distinct experiments). *D*, line plot pixel intensity for RFP (*magenta*) and GFP (*green*) for lines in (*C*). *E*, quantification of the transiently expressed GFP-ezrin_1-586_ (WT or T567D mutant) peripheral: cytoplasmic pixel intensity in the presence and absence of transiently expressed RFP-CLIC5A. *Top panel:* each data point represents a single cell from a single experiment (two-way ANOVA: interaction F = 19.3, *p* < 0.0001; CLIC5A effect F = 23.6, *p* < 0.0001; ezrin mutant *versus* WT effect F = 156.9; *p* < 0.0001, *p* values for *posthoc* Tukey’s multiple comparisons are shown). *Bottom panel:* each data point represents the mean for one of three biologically independent experiments. (Two-way ANOVA: interaction F = 28.8, *p* < 0.0007; CLIC5A effect F = 32.7, *p* < 0.0004; ezrin mutant *versus* WT effect F = 97.3; *p* < 0.0001, *p* values for *post hoc* Tukey’s multiple comparisons are shown). *F*, quantification of the RFP-CLIC5A peripheral: cytoplasmic pixel intensity in the presence or absence of transiently expressed GFP-ezrin1-586 (WT or T567D mutant). *Top panel:* each datapoint represents a single cell from a single experiment (one-way ANOVA F = 0.297, *p* = 0.744). *Bottom panel:* each data point represents the mean for one of three biologically independent experiments (one-way ANOVA F = 0.796, *p* = 0.488). *G*, Pearson correlation coefficient for colocalization of transiently expressed RFP-CLIC5A and GFP-ezrin (WT or T567D mutant). *Right panel:* each data point represents a separate image (each image containing 4–10 cells) from a single experiment (two-way ANOVA: interaction F = 13.51, *p* < 0.0007; separate *versus* cotransfection F = 1175, *p* < 0.0001; ezrin mutant *versus* WT effect F = 22.54; *p* < 0.0001, *p* values for *post hoc* Tukey’s multiple comparisons are shown). *Right panel:* each datapoint represents the mean from one of three biologically distinct experiments (mean ± SD, Student’s *t* test). CLIC, chloride intracellular channel; ERM, ezrin, radixin, and moesin; GST, glutathione S-transferase; WB, Western blot.

Since active ERM proteins are phosphorylated at a highly conserved C-terminal Thr residue (T567 in human ezrin), we explored whether ezrin phosphorylation alters its interaction with CLIC5A. Treatment of COS-7 cells with the Ser/Thr phosphatase inhibitor calyculin-A for 15 min prior to lysis significantly increased the pulldown of transiently expressed GFP-ezrin_432-586_ by GST-CLIC5A from the lysates (Fig. 4*A*). Endogenous ezrin was also pulled down more effectively by GST-CLIC5A from cells treated with calyculin-A compared to cells not treated with calyculin A (Fig. 4*A*). Similarly, ezrin_432-586_ with a T567D phosphomimetic point mutation was pulled from cell lysates by GST-CLIC5A more effectively than transiently expressed WT or phosphorylation deficient T567A mutant ezrin_432-586_ fragments (Fig. 4*B*). These observations suggest that CLIC5A binding to the ezrin C terminus is enhanced by T567 phosphorylation.

We next determined the impact of CLIC5A expression on the localization of transiently expressed full-length GFP-ezrin_1-586_ with and without a phosphomimetic T567D point mutation in living HeLa cells. We were surprised to find that expressed WT GFP-ezrin_1-586_ was predominantly cytosolic without significant accumulation at the cell periphery (Fig. 4, *C*–*E*). A cytosolic distribution of expressed full-length ezrin was previously described by Auvinen *et al.* (30). By contrast, transiently expressed GFP-ezrin_1-586_ harboring the T567D phosphomimetic point mutation, localized more effectively to the cell periphery than WT GFP-ezrin_1-586_ (Fig. 4, *C*–*E*). That ezrin phosphorylation at T567 results in ezrin targeting to cortical actin without the necessity of PI(4,5)P_2_ binding was previously shown by Fievet *et al.* (31). We also observed that transient coexpression of RFP-CLIC5A with GFP-ezrin significantly shifted the phosphomimetic GFP-ezrin_1-586_ (T567D) to the cell periphery but did not alter the peripheral localization of WT GFP-ezrin_1-586_ (Fig. 4, *C*–*E*). The RFP-CLIC5A peripheral:cytosolic ratio was not changed by overexpression of WT or phosphomimetic GFP-ezrin_1-586_ (Fig. 4*F*). In keeping with these findings, the Pearson correlation coefficient indicated that there was greater colocalization of RFP-CLIC5A with the phosphomimetic GFP-ezrin_1-586_ (T567D) mutant than with WT GFP-ezrin_1-586_. Taken together, these results indicate that ezrin phosphorylation at T567 increases its affinity for CLIC5A and imply that ezrin phosphorylation enhances its interaction with CLIC5A at the cell periphery.

### Indirect interaction between CLIC5A and Rac-GTP

**Figure 5:**
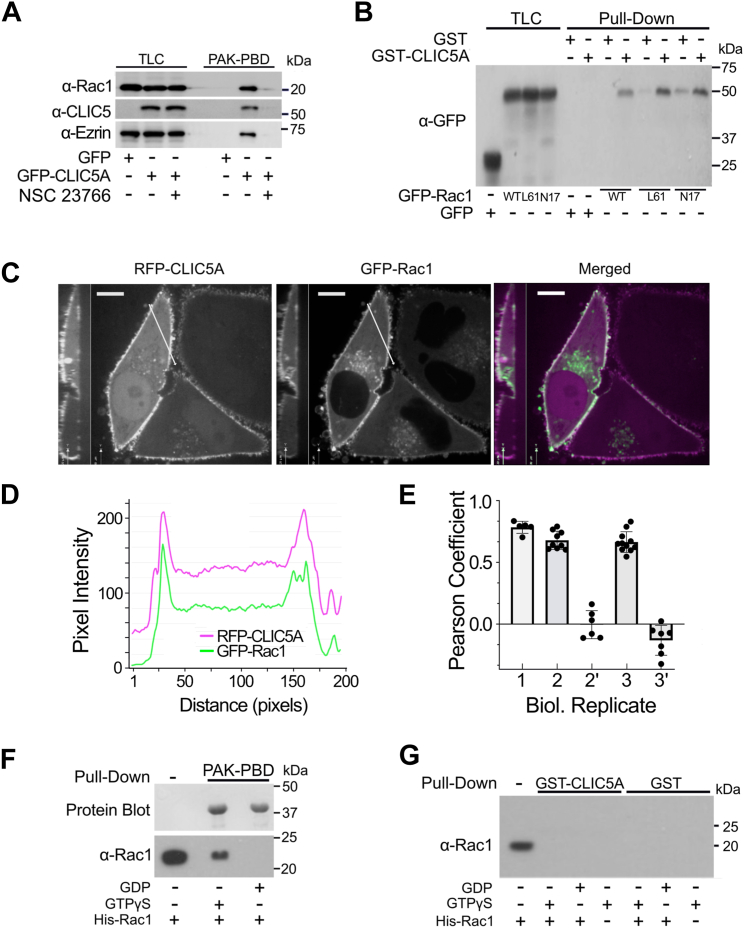
**Indirect association of CLIC5A and Rac1.***A*, WB showing endogenous Rac1 and ezrin as well as transiently expressed GFP-CLIC5A in total cell lysates (TLC) and the same proteins captured by immobilized PAK-protein binding domain (PAK-PBD) from HeLa cells transiently expressing GFP or GFP-CLIC5A. The GFP-CLIC5A expressing cells were treated, or not, with the Rac1 inhibitor NSC23766 (100 μM × 10 min) (representative of three biologically distinct experiments). *B*, α-GFP WB of GST- or GST-CLIC5A pulldowns from lysates of COS-7 cells transiently expressing WT, constitutively active (L61) or dominant negative (N17) GFP-Rac1 constructs (representative of three independent experiments). *C*, live-cell imaging of HeLa cells transiently expressing RFP-CLIC5A (magenta) and WT GFP-Rac1 (*green*). The scale bar represents 10 μm. The *white lines* represent line plots analyzed in (*D*). Colocalization (*white*) in the merged image is observed at the dorsal cell periphery and in the perinuclear location (representative of three biologically distinct experiments). *D*, pixel intensity of line plots in (*C*) for RFP-CLIC5A (*magenta*) and GFP-Rac1 (*green*). *E*, Pearson correlation coefficients of all images from three biologically distinct experiments. 1, 2, and 3 = cotransfection of GFP-Rac1 with RFP-CLIC5A; 2’ and 3’ = separate transfection of GFP-Rac1 or RFP-CLIC5A followed by 1:1 mixing of cells. *F*, *in vitro* loading of purified, recombinant His-Rac1 with GTPγS *in vitro*. His-Rac1 (500 ng) was incubated with GDP or GTPγS followed by affinity capture of Rac1-GTPγS by immobilized PAK-PBD. *Top panel:* protein blot. *Bottom panel:* α-Rac1 WB. *G*, α-Rac1 WB of purified His-Rac1 and GST- or GST-CLIC5A pulldowns from cell-free solutions containing WT Rac1, Rac1-GTPγS, or Rac1-GDP (representative of three independent experiments). CLIC, chloride intracellular channel; GST, glutathione S-transferase; WB, Western blot.

CLIC5A expression augments Rac1 activity in COS-7 cells (24), and Rac1 activity is enhanced through sequestration of the Rho-GDI by ezrin (32). To examine the relationship between ezrin, CLIC5A, and Rac1, we first determined whether CLIC5A and ezrin coprecipitate with Rac1-GTP pulled from cells. HeLa cells were transfected with *GFP*-vector or *GFP-CLIC5A* cDNAs and treated, or not, with the Rac1 inhibitor NSC 23766 (100 μM, 10 min), followed by pulldown with immobilized PAK-protein binding domain (PAK-PBD), to capture active Rac1-GTP. Transient GFP-CLIC5A expression raised Rac1-GTP levels, consistent with our previous findings (24), and both, GFP-CLIC5A and endogenous ezrin coprecipitated with endogenous Rac1-GTP. When Rac-GTP generation was inhibited by NSC 23766 neither endogenous ezrin nor transiently expressed CLIC5A were brought down by PAK-PBD beads (Fig. 5*A*). We therefore wondered whether Rac1-GTP might interact directly with CLIC5A. WT GFP-Rac1, constitutively active GFP-Rac1 Q61L, and dominant negative GFP-Rac1 D17N transiently expressed in COS-7 cells were all pulled from cell lysates by GST-CLIC5A without apparent preference for constitutively active GFP-Rac1 (Fig. 5*B*). Live-cell imaging of HeLa cells cotransfected with RFP-CLIC5A and WT GFP-Rac1 showed that RFP-CLIC5A colocalized with GFP-Rac1at the cell periphery (Fig. 5*C*), demonstrated by the intensity line plots showing overlapping peaks for GFP-Rac1 and RFP-CLIC5A (Fig. 5*D*). Pearson coefficients for GFP-Rac1/RFP-CLIC5A colocalization determined for three biologically distinct experiments averaged 0.74 ± 0.05, 0.68 ± 0.07, and 0.67 ± 0.08 (n = 5, 9, and 11 images, respectively, mean ± SD, Fig. 5*E*), consistent with colocalization (Fig. 5*E*). In two biologically distinct control experiments cells were transfected separately with GFP-Rac-1 or RFP-CLIC5A cDNAs and then mixed 1:1 followed by imaging. The Pearson correlation coefficients for these controls were −0.005 ± 0.11 and −0.13 ± 0.12 (n = 6 and 7 images, respectively; Fig. 5*E*). Nonetheless, even though Rac1-GTP and CLIC5A coprecipitated and colocalized at the cell periphery, purified, recombinant His-Rac1 loaded with GTPγS or GDP (Fig. 5*F*) did not interact with purified GST-CLIC5A *in vitro* (Fig. 5*G*). Therefore, in the presence of transiently expressed GFP-CLIC5A, endogenous ezrin, endogenous Rac1-GTP, and GFP-CLIC5A become part of the same protein complex at the cell periphery, but the interaction between CLIC5A and Rac-GTP appears to be indirect.

### Functional consequences of reducing the CLIC5A/ezrin interaction

**Figure 6:**
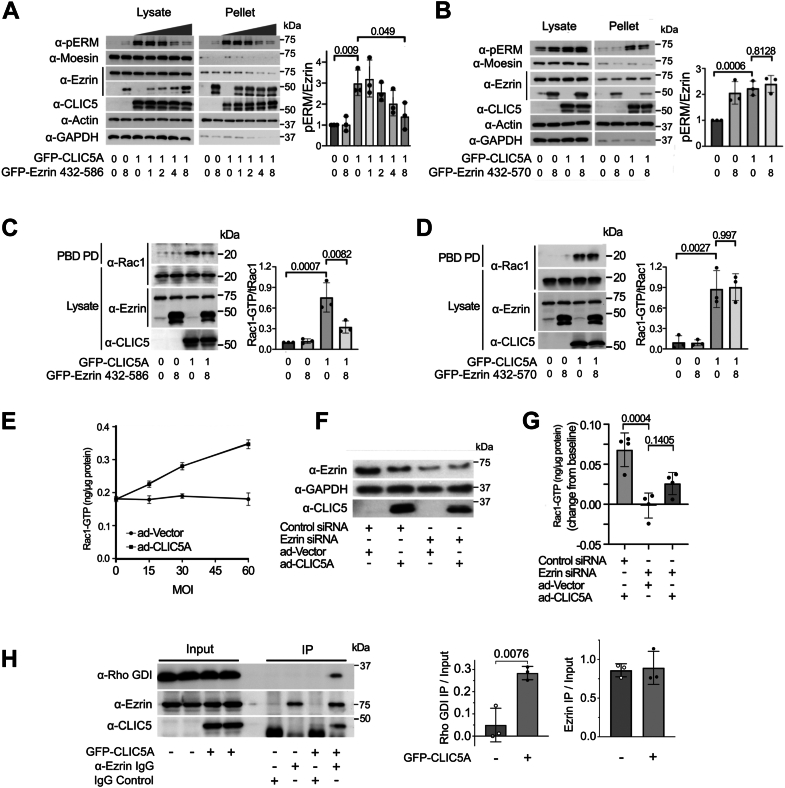
**ERM and Rac-1 activation are amplified by the CLIC5A/ezrin interaction.***A*, endogenous pERM abundance in lysates and detergent resistant pellets of COS-7 cells transfected with *GFP-CLIC5A* cDNA and increasing concentrations of *GFP-ezrin*_*432-586*_*(T567D)cDNA*. *GFP-CLIC5A* cDNA was kept constant and the *GFP-CLIC5A:GFP-ezrin*_*432-586*_*(T567D)* cDNA transfection ratio was 1:1, 1:2, 1:4, and 1:8. *Left panel*, representative WB. *Right panel,* densitometric quantification of the endogenous pERM: endogenous ezrin ratio (mean ± SD, n = 3 independent experiments, one-way ANOVA: F = 7.90; *p* = 0.0007. *p* values shown represent *post hoc* Dunnett’s multiple comparisons). *B*, pERM abundance in lysates and detergent resistant pellets of COS-7 cells transiently transfected with *GFP-CLIC5A* cDNA with or without an 8-fold excess of *GFP-ezrin*_*432-570*_ cDNA. *Left panel*, representative WB. *Right panel,* densitometric quantification of the endogenous pERM: endogenous ezrin ratio (mean ± S.D., n = 3 biologically independent experiments, two-way ANOVA: interaction F = 12.25; *p* = 0.01; CLIC5A effect: F = 39.63, *p* = 0.0002; ezrin_432-570_ effect F = 22.58. *p* = 0.0014; *p* values shown represent *post hoc* Tukey’s multiple comparisons). *C*, endogenous Rac1 WB for COS-7 cell lysates and endogenous Rac1-GTP captured by PAK-PBD pulldown (PD) from COS-7 cells transiently transfected with *GFP-CLIC5A* cDNA with or without an 8-fold excess of *GFP-ezrin*_*432-586*_*(T567D)* cDNA. *Left panel,* representative WB. *Right panel:* densitometric quantification of endogenous Rac1-GTP/total endogenous Rac1 (mean ± SD, n = 3 independent experiments, two-way ANOVA: interaction F = 6.32, *p* = 0.036; CLIC5A effect F = 29.25, *p* = 0.001; ezrin 432–586 effect F = 5.88; *p* = 0.042, *p* values for *post hoc* Tukey’s multiple comparisons are shown). *D*, PAK-PBD pulldown (PD) of endogenous Rac1-GTP from lysates of COS-7 cells transfected with *GFP-CLIC5A* cDNA with or without an 8-fold excess of transiently expressed *GFP-ezrin432-570* cDNA. *Left panel,* representative WB. *Right panel,* densitometric quantification of endogenous Rac1-GTP/total endogenous Rac1 (mean ± S.D., n = 3 independent experiments, two-way ANOVA: interaction F = 0.029, *p* = 0.87; CLIC5A effect F = 62.17 *p* < 0.0001; ezrin 432–570 effect F = 0.012; *p* = 0.91, *p* values for *post hoc* Tukey’s multiple comparisons are shown). *E*, endogenous Rac1-GTP abundance in human glomerular endothelial cells (hGENs) determined by Rac1-GTP G-LISA. The cells were transduced with control adenoviral-vector (ad-Vector) or untagged *CLIC5A* cDNA in the same vector (ad-*CLIC5A*) at an increasing multiplicity of infection (MOI) (mean ± SD, n = 3 independent experiments). *F*, representative WB of lysates from hGEN cells transduced with ad-Vector or ad-*CLIC5A* with or without ezrin-specific siRNA. *G*, change relative to baseline of Rac1-GTP in hGEN cells transduced with 30 MOI ad-Vector or ad-*CLIC5A* with or without ezrin-specific siRNA (mean ± SD, n = 4 independent experiments, two-way ANOVA: interaction F = 6.35, *p* = 0.027; CLIC5A effect F = 34.18, *p* < 0.0001; ezrin siRNA effect F = 6.86; *p* = 0.023, *p* values shown represent *post hoc* Tukey’s multiple comparisons). *H*, coimmunoprecipitation of endogenous Rho-GDI with endogenous ezrin in the presence and absence of transiently expressed GFP-CLIC5A. *Left panel:* representative α-Rho GDI, α-ezrin, and α-CLIC5A WB of lysates (input) and α-ezrin immunoprecipitates (IP). *Right panel:* Quantification of endogenous Rho-GDI and endogenous ezrin immunoprecipitated with α-ezrin antibodies (n = 3 biologically distinct experiments, mean ± SD, Student’s *t* test). CLIC, chloride intracellular channel; PBD, protein binding domain; pERM, phosphorylated ezrin, radixin, and moesin proteins; Rho-GDI, Rho guanine nucleotide dissociation inhibitor; WB, Western blot.

Since the phosphomimic ezrin_432-586_ (T567D) fragment binds CLIC5A, we reasoned that its expression in cells might competitively inhibit CLIC5A-dependent functions. *GFP-CLIC5A* cDNA was transfected at a ratio of 1 μg cDNA: ∼10^6^ COS-7 cells, together with GFP-ezrin_432-586_ (T567D) at concentrations ranging from 0 to 8 μg cDNA per ∼10^6^ cells. Overexpressed GFP-ezrin_432-586_ (T567D) was found predominantly in the insoluble “cytoskeletal” fraction (Fig. 6*A*). CLIC5A-dependent phosphorylation of endogenous ERM proteins was inhibited by GFP-ezrin_432-586_ (T567D) in a concentration-dependent fashion, as was the association of endogenous ezrin with the insoluble “cytoskeletal” fraction (Fig. 6*A*). By contrast, coexpression of an 8-fold excess GFP-ezrin_432-570_, which does not bind CLIC5A, failed to reduce CLIC5A-stimulated ERM phosphorylation and did not inhibit its association with the cytoskeletal fraction (Fig. 6*B*). When GFP-ezrin_432-586_ (T567D) was expressed at an 8-fold excess over GFP-CLIC5A, CLIC5A-stimulated Rac1-GTP generation was significantly blunted (Fig. 6*C*), while GFP-ezrin_432-570_ did not inhibit CLIC5A-stimulated Rac1 activation (Fig. 6*D*). Actin- and PI(4,5)P_2_-associated ERM proteins in their unfolded, active form were previously shown to sequester Rho-GDI resulting in Rac1 activation (32). We therefore determined whether CLIC5A-dependent Rac1 activation requires ezrin. Transient expression of untagged CLIC5A from an adenoviral construct increased Rac1-GTP levels in a concentration-dependent fashion in human glomerular endothelial cells (Fig. 6*E*), similar to the findings in HeLa cells (Fig. 5*A*) and COS-7 cells (24). siRNA-mediated knockdown of ezrin in the endothelial cells (Fig. 6*F*) significantly blunted CLIC5A-stimulated Rac1 activation (Fig. 6*G*). Finally, immunoprecipitation of endogenous ezrin coprecipitated significantly more Rho-GDIα from COS-7 cells transiently expressing CLIC5A, compared to controls not expressing CLIC5A (Fig. 6*H*). Taken together, the findings indicate that the interaction of CLIC5A with ezrin is required for the functional effects of CLIC5A on ERM phosphorylation and Rac1 activation and that CLIC5A-stimulated Rac-GTP generation is mediated, at least in part, by sequestration of Rho-GDIα by active ezrin.

## Discussion

CLICs were originally named “chloride intracellular channels” based on their tendency to partition into artificial lipid bilayers, thus facilitating IAA-94 inhibitable Cl^−^ movement across lipid bilayers down concentration gradients. Indeed, several recent reviews still assign Cl^−^ channel function to these proteins (33, 34, 35). Endogenous CLIC5A does localize to the apical membrane of placental trophoblasts (7) and to the apical domain of podocyte foot processes (17), and in COS-7 cells ectopically expressed GFP-CLIC5A colocalizes with PI(4,5)P_2_ and PI4P5 kinase at the dorsal plasma membrane (25), which could all be consistent with a plasma membrane channel. However, CLIC crystal structures predict that they are soluble proteins. Here, we observed that although CLIC5A concentrations are highest at the cell periphery (Figure 3, Figure 4, Figure 5), the majority of ectopically expressed CLIC5A in COS-7 cells is soluble, is not associated with the lipid membrane fraction and is not detected at the cell surface by biotinylation, protease susceptibility or immunoreactivity (Fig. 1). These findings indicate that transiently expressed CLIC5A is not a membrane-spanning protein and therefore cannot function as an ion channel. It could be argued that ectopically expressed CLIC5A does not necessarily behave like endogenous CLIC proteins. However, cell fractionation experiments showed that endogenous CLIC1 and CLIC4, which are highly homologous with CLIC5A, are also soluble (Fig. S2) (28). It could still be argued that digitonin disrupts a direct association of CLIC5A, CLIC4, and CLIC1 with the inner leaflet of the plasma membrane through depletion of cholesterol (36). In fact, association of CLIC1 with lipid bilayers was previously reported to be cholesterol-dependent (37). However, in cells, cholesterol is found predominantly in the outer leaflet of the cell membrane, making this possibility unlikely (38). The previous findings by Berryman *et al.* (11) that despite its location at the apical plasma membrane, expressed CLIC5A does not alter Cl^−^ efflux together with the current studies showing that CLIC5A is not an integral membrane-spanning protein, we can confidently conclude that CLIC5A is not a membrane-spanning Cl^−^ channel.

It was already known that CLIC5A is part of the ezrin complex in glomerular podocytes (17) and placental microvilli (7), and that it is part of the radixin complex in inner ear sensory stereocilia (18). However, a direct interaction between ERM proteins and CLIC5A was not previously reported. An unbiased Y2H protein domain screen, performed for us by Hybrigenics (Evry) suggested that the radixin C-terminal tail can interact directly with CLIC5A. This finding was promising since a functional interaction of radixin and CLIC5A in the inner ear hair cells was previously established (18). Here, we show that CLIC5A interacts directly with the ERM C-terminal tails, though the interaction with ezrin appears to be the strongest, at least by Y2H assay. The direct interaction between CLIC5A and ezrin is in keeping with the original observation by Berryman *et al.* (7), who discovered CLIC5A in a cytoskeletal protein complex precipitated with an immobilized 36-aa ezrin C-terminal peptide. Nonetheless, it is of note that the interaction of CLIC5A with the ezrin C terminus while strengthened by phosphorylation at T567 has a relatively modest affinity in the 30 μM range when the purified proteins are coincubated. This is in keeping with the observation that CLIC5A is largely soluble in digitonin permeabilized cells (Fig. 1). The reported K_d_ for the high affinity autoinhibitory interaction between the ezrin N-terminal FERM domain and its C-terminal tail is 176 nM (39). Based on these affinity differences, it follows that direct binding by CLIC5A would not independently activate ezrin, implying that ezrin activation by membrane PI(4,5)P_2_, actin, and T567 phosphorylation (40) precedes CLIC5A binding. Relatively weak interactions can be physiologically important, like those involving SH3 domains which typically are in the range of 1 to 200 μM (41). For CLIC5A, expression in kidney podocytes is extremely high reaching levels nearly 3 orders of magnitude greater than that in most other cells (17, 42, 43) so it seems plausible that localized concentrations reach 30 μM or greater. It is also possible that the interaction of CLIC5A with ezrin within the cell is greater than that of purified GST-CLIC5A for the ezrin_482-586_ fragment *in vitro* (Fig. 2*D*). Activated ezrin exists in a complex with actin and actin-associated proteins. In this regard, CLIC5A/ezrin binding is completely abolished by deletion of the C-terminal 16 aa of ezrin (Fig. 2, *E*–*G*), which would also disrupt the ezrin/actin interaction (44), and T567 ezrin phosphorylation enhances ezrin association with actin (45) as well as its interaction with CLIC5A (Fig. 4). Y2H mapping furthermore indicates that CLIC5A ezrin binding requires the short 16-aa ezrin_570-586_ actin binding motif (Fig. 2*C*) (44), and Berryman and Bretscher (7) found that the association of CLIC5A with the C-terminal 36 aa of ezrin is facilitated by actin. Therefore, the affinity of CLIC5A for ezrin may be enhanced by the ezrin–actin interaction, and it is conceivable that CLIC5A, ezrin, and actin form a more stable complex under physiological conditions. Further studies are required to address these possibilities.

We postulated that ezrin might recruit CLIC5A to the apical subplasma membrane space. However, ezrin silencing in HeLa cells had only a minor and inconclusive effect on CLIC5A localization (data not shown). But when ERM proteins were silenced simultaneously, a significant portion of GFP-CLIC5A failed to localize to the cell periphery (Fig. 3), indicating that the interaction of CLIC5A with its ERM partners is partly responsible for its peripheral localization. Even so, there still was substantial peripheral GFP-CLIC5A when ERM proteins were silenced. This could be due to residual ERM protein expression, but additional mechanisms may also target CLIC5A to its peripheral location. We know that CLIC5A induces actin polymerization and remodeling of the dorsal cell surface in COS-7 cells raising the possibility that CLIC5A interacts directly with polymerized/filamentous actin or other constituents of the cortical actin cytoskeleton. CLIC1 and CLIC4 are similarly recruited to areas of active actin remodeling, such as filipodia (46, 47) and the cytokinesis cleavage furrow (48), and they associate with other actin-associated proteins (48, 49). Among these, the actin-associated protein phosphatase-1 regulatory protein, taperin (50) interacts directly with several CLIC homologs (51). Taperin is relevant to CLIC5A because both localize to the tapered base of inner ear hair cell stereocilia (23) and both are implicated in familial nonsyndromic deafness. So, localization of CLIC5A to the cortical actin cytoskeleton depends partly on ERM proteins, but other actin partners likely also play a rule.

**Figure 7:**
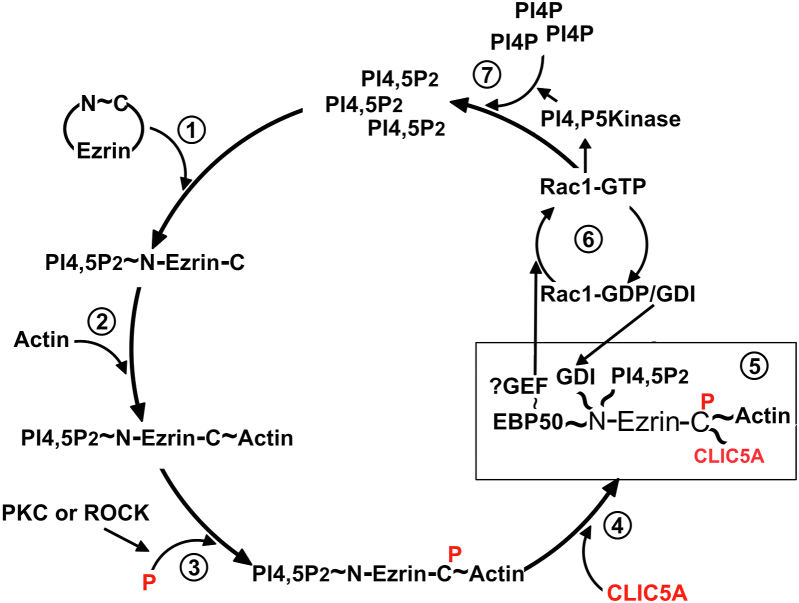
**CLIC5A-dependent feed-forward ezrin activation loop.***1*, the soluble (inactive) conformation of ezrin, in which ezrin N and C termini self-associate with high affinity encounters plasma membrane PI(4,5)P_2_. *2*, a conformational change brought about by binding of the ezrin N-terminal FERM domain to membrane PI(4,5)P_2_ unmasks the ezrin C-terminal domain which binds filamentous actin producing the open/active conformation of ezrin. *3*, this open ezrin conformation is subject to phosphorylation of T567 by PKC or Rho-dependent kinase (ROCK) enhancing actin binding. *4*, the actin-associated phosphorylated ezrin C terminus directly binds CLIC5A. *5*, CLIC5A binding stabilizes the open/active conformation of ezrin. *6*, open/active ezrin sequesters inhibitory Rho-GDI (GDI), removing it from Rac-GDP and resulting in spatially restricted Rac1-GTP generation. Open/active ezrin also binds NHERF1 (EBP50) and NHERF2. EBP50 can recruit the Rac GEF ARHGEF7 also known as β-Pix (57, 58), which would also enhance Rac1-GTP formation in the absence of the GDI. Rac1-GTP is known to stimulate the localized generation of PI(4,5)P_2_ by PI4P5 kinases. By stabilizing the active ezrin hub, CLIC5A promotes Rac1-GTP–stimulated accumulation of PI(4,5)P_2_ which then serves to further activate ezrin. Thus, the interaction of CLIC5A with the open/active conformation of ezrin results in feed-forward amplification loop of ezrin-stimulated Rac1 activity and PI(4,5)P_2_ generation, in turn serving to stabilize the local cortical actin cytoskeleton. CLIC, chloride intracellular channel; PI(4,5)P_2_, phosphatidylinositol 4,5 bisphosphate; Rho-GDI, Rho guanine nucleotide dissociation inhibitor.

The observation that full-length ezrin does not interact with CLIC5A in the Y2H or the GST-CLIC5A pull-down assays is most consistent with autoinhibition of soluble ezrin due to the intramolecular interaction between the ezrin N-terminal FERM domain and its C-terminal CERMAD domain (29). This N-/C-terminal interaction is dynamic and complex (39) and makes the C-terminal tail of ezrin unavailable for CLIC5A binding. However, we were able to pull endogenous full-length ezrin from cell lysates with GST-CLIC5A when dephosphorylation was inhibited (Fig. 4*A*). Also, HA-ezrin and GFP-CLIC5A were coimmunoprecipitated when both proteins were coexpressed in cells. The findings imply that full-length ezrin_1-586_ can interact with CLIC5A when the ezrin C-terminal ERMAD domain is free of the interaction with its own FERM domain (Fig. 7) (29). Ezrin phosphorylation at T567, located just 19 residues from the ezrin C-terminal end, has long been established as a component of ezrin activation, promoting the interaction of the ezrin C terminus with filamentous actin. We observed that the CLIC5A interaction with the ezrin C terminus increases when ezrin is phosphorylated at T567 (Fig. 4). The finding that the peripheral localization of the ezrin_1-586_ phosphomimetic T567D mutant increases in the presence of CLIC5A, whereas CLIC5A does not alter the largely cytosolic location of WT ezrin_1-586_ (Fig. 4) implies that in cells ezrin must be in the active, open conformation and phosphorylated, and probably bound to cortical actin, for a functionally significant interaction with CLIC5A (Fig. 7).

Previous findings that CLIC5A expression leads to enhanced Rac1 activation (24) led us to determine whether CLIC5A interacts directly with Rac1. Interestingly, both transiently expressed CLIC5A and endogenous ezrin were pulled from cell lysates by Rac-GTP in the conventional PAK-PBD pull-down assay (Fig. 5*A*), which precipitates Rac1-GTP, but not Rac-GDP. RFP-CLIC5A also colocalized strongly with transiently expressed GFP-Rac1 at the cell periphery (Fig. 5*C*). But even though Rac1 and CLIC5A coprecipitated and colocalized in cells, a direct interaction between CLIC5A and Rac1, whether loaded with GTP or not, was not observed (Fig. 5*G*) *in vitro* and in one pilot experiment purified GST-CLIC5A did not have Rac-GEF activity *in vitro* (data not shown). A direct effect of CLIC5A on Rac1 activity is therefore highly unlikely.

The findings that overexpression of the ezrin_432-586_ fragment with the T567D mutation inhibited ERM phosphorylation in a dose-dependent fashion and as well as Rac1 activation (Fig. 6, *A*–*D*), and that CLIC5A-dependent Rac1 in activation in endothelial cells was ezrin-dependent (Fig. 6*G*) suggested that the interaction of CLIC5A with ezrin indirectly activates Rac1. It is well-established that activated ERM proteins directly bind the Rho-GDP dissociation inhibitor alpha (ARHGDIA) (32). Rho-GDI interacts with the ezrin N-terminal FERM domain, but only if it is not occupied by its own C-terminal CERMAD domain (52). Here, expression of CLIC5A significantly increased Rho-GDIα coimmunoprecipitation with endogenous ezrin (Fig. 6*H*), implying that the interaction of CLIC5A with the ezrin C-terminal domain increases Rac1 activity, at least in part, by augmenting the sequestration of Rho-GDIα by ezrin. The conclusion that CLIC5A-stimulated Rac1 activation is ezrin-mediated could have been further strengthened by demonstrating rescue with overexpressed ezrin, which was not done here.

A recognized limitation of these mechanistic studies in cells is the fact that ectopic expression of proteins can lead to spurious results, and GFP or RFP fusion proteins can lead to mislocalization of proteins. CLIC5A overexpression was necessary as all cultured cells we examined (43), including cultured podocytes, express little or no CLIC5A at baseline (data not shown). The fact that RFP and GFP controls did not localize to the cell periphery suggests that RFP and GFP were not responsible for the membrane/peripheral localization of GFP-CLIC5A, RFP-CLIC5A, GFP-ezrin, or GFP-Rac1. Furthermore, endogenous CLIC5A and ezrin localize to the plasma membrane at the apical domain of podocytes foot processes *in vivo* (17). We have also previously documented that CLIC5 deletion *in vivo* profoundly reduces ERM phosphorylation and activation of the Rac1 effector PAK1/3 (24). Despite the limitations, we expect that mechanisms observed here will largely hold when further studies are done with endogenous CLIC5A.

To sum up, CLIC5A, previously known to be a component of ERM protein complexes in inner ear hair cells and podocytes *in vivo*, interacts *directly* with the C-terminal domains of ERM. Transiently expressed CLIC5A does not display characteristics that would be expected of a membrane-spanning channel. The direct interaction of ezrin with CLIC5A requires the open/active conformation of ezrin, which is augmented by ezrin phosphorylation at the C-terminal T567 and promotes Rac1 activation through enhanced sequestration of Rho-GDI by ezrin. The data best fit a model in which ezrin activation by PI(4,5)P_2_ and actin is required as a first step, followed by association of CLIC5A with the ezrin C-terminus and consequent activation of Rac1 by sequestering inhibitory Rho-GDI (Fig. 7). We postulate that CLIC5A, by binding directly to ezrin, stabilizes the ezrin open/active conformation, and potentially its interaction with actin. This would promote a feed-forward amplification loop of localized Rac1 activity, driving local plasma membrane PI(4,5)P_2_ generation (25) in turn further increasing localized ezrin docking and activation. PI(4,5)P_2_ is a recognized hub for localized actin remodeling (53) in keeping with CLIC5A-stimulated actin polymerization (25). This mechanism plays a role in the formation of placental microvilli and seems to be indispensable for the long-term maintenance of inner ear actin-based stereocilia and podocyte foot processes in kidney glomerular capillaries.

## Experimental procedures

### Reagents and antibodies

Reagents were purchased from Sigma-Aldrich Canada Co. unless otherwise stated. The Matchmaker Gold Y2H system was from Takara Bio USA (# 630489). The siRNAs targeting ezrin in HeLa cells (#SR305077), radixin (#SR304025), and moesin (#SR305077) were purchased from OriGene Technologies, Inc. The siRNA-targeting human ezrin in glomerular endothelial cells (#sc-37007) was from Santa Cruz Biotechnology. Calyculin-A and m-3M3FBS were from Millipore (Billerica). The source of antibodies and dilutions used are provided as supporting information (Tables S1 and S2).

### Cell culture and transfection

COS-7 and HeLa cells were cultured in Dulbecco’s modified Eagle medium (Thermo Fisher Scientific) with 10% *v*/*v* fetal bovine serum (FBS; Life Technologies) and 1% *v*/*v* penicillin/streptomycin (Life Technologies) at 37 °C in humidified air containing 5% CO_2_. Mycoplasma-free primary human glomerular endothelial cells (hGENs) were purchased from Angio-Proteomie (# cAP-0004) and maintained in EGM-2 MV Bulletkit growth media (# CC-3162, Lonza) containing 5% FBS and 1% penicillin/streptomycin at 37 °C in humidified air containing 5% CO_2_. Cells were cultured on Quick Coating Solution (Angio-Proteomie, # cAP-01). Cells in the logarithmic phase of growth were transfected using Lipofectamine 2000 (Invitrogen, # 11668-027) according to the manufacturer’s protocol. For COS-7 and HeLa cells, CLIC5A plasmid was used at a concentration of 2 μg/35 mm culture plate (∼10^6^ cells) unless otherwise stated. This achieved a level of CLIC5A protein expression that was similar to that in mouse glomeruli *in vivo* (Fig. S1). Adenoviral vector pAdTrack-GFP/CLIC5A expressing GFP and CLIC5A from separate promoters and pAdTrack-GFP were used to transduce cells at approximately 80% confluence in 1 ml medium containing 5 μg/ml polybrene (EMD Millipore). Human ezrin-specific siRNA and nonspecific control siRNA were transfected into ∼70% confluent hGEN in 35-mm plates using 1.0 ml antibiotic-free EBM-2 medium mixed with 5 μl Lipofectamine 3000 in 200 μl of Opti-MEM I medium (Life Technologies) to achieve a final siRNA concentration of 10 nM. Six hours later, 1.0 ml antibiotic-free EBM-2–containing 10% FBS was added. Twenty-four hours after addition of siRNA, the medium was changed to complete EGM-2 containing growth factors, antibiotics, and 5% FBS. Human ezrin- radixin- and/or moesin siRNA were transfected into HeLa cells using the same protocol but grown in Dulbecco’s modified Eagle medium containing 10% FBS and antibiotics.

Forty-eight hours after transfection, addition of siRNA or transduction with adenoviral vectors, cells were washed with ice-cold PBS and lysates were prepared with cell lysis buffer (# GL36, Cytoskeleton Inc.) containing protease inhibitor cocktail (# PIC02, Cytoskeleton Inc.) and phosphatase inhibitor (#04906837001, Roche).

### Cloning and generation of vector constructs

The full-length human CLIC5A coding region in the pCDNA3.1-vector was originally prepared from a human kidney cDNA library (17) and served as the template for all CLIC5A constructs in this study. N-terminal FLAG-, GFP-, and GST-epitope tagged CLIC5A fusion constructs were prepared in pTARGET (#TM044, Promega), pEGFP-C1 (#6084-1, Clontech Laboratorie), and pGEX-3 (GE Healthcare) vectors, respectively. Plasmids were reproduced in *Escherichia coli* DH5α using the QIAgen MaxiPrep kit. The pGEX-3-GST or pGEX-3-GST-CLIC5A constructs were transformed into *E. coli* BL21 Gold (DE3) (Agilent Technologies, Santa Clara), followed by induction with 0.2 mM IPTG and grown at 37 °C for 4 h. Bacteria were harvested in PBS containing 1× proteinase inhibitor cocktail (Roche), followed by sonication and addition of 1% Triton X-100. Affinity chromatography-based purification of GST, and GST-CLIC5A proteins on glutathione-Sepharose 4B (GE Healthcare) was done as per manufacturer’s instructions. The GFP- and HA-human ezrin cDNA fusion constructs were prepared in pEGFP-C1 and pTARGET vectors, respectively. For the Y2H assay, full-length ezrin, ezrin fragments as well as radixin and moesin fragments (Fig. 2*C*) were cloned into the pGAD-T7 prey vector and full-length CLIC5A and CLIC5A fragments were cloned into the pGBKT bait vector. For all constructs, sequence orientation and fidelity were confirmed by restriction digestion and sequencing of the inserts.

### Microscale thermophoresis

Expression plasmids for GST-His_6x_-CLIC5A and His_6x_-PagP-ezrin_482-586_ fusion constructs were created by ATUM (formerly DNA 2.0). To facilitate inclusion body formation in *E. coli* BL21 (DE3), the ezrin construct contains an N-terminal PagP fusion partner. Since ezrin_432-586_ contains a natural internal Ni^2+^-sensitive cleavage site (S482YHV), we used this cleavage site to separate ezrin_482-586_ from PagP, which was then utilized for MST. As the ezrin_482-586_ sequence contains an additional intrinsic Ni^2+^-sensitive cleavage site at H549, an H549N substitution was also made. To enhance CLIC5A binding, the ezrin_482-586_ construct also contains a T567E phosphomimetic mutation. The fusion protein was expressed in *E. coli* BL21 (DE3) cells, purified by nickel-nitriloacetic acid affinity chromatography in 6M guanidine hydrochloride. The PagP fusion partner was removed using Ni^2+^-catalyzed peptide bond cleavage as previously described (54).

MST measures the rate of diffusion of fluorescently labeled proteins through a microscopic temperature gradient induced by infrared laser pulses. Interactions between fluorescently labeled target and unlabeled ligand proteins slow the thermophoretic movement resulting in changes of the fluorescence signal. MST was performed using NanoTemper Monolith NT.115 (NanoTemper Technologies). Purified GST-His_6x_-CLIC5A and GST-His_6x_ proteins and a His_6x_ control peptide were labeled with the Monolith His-Tag Labeling Kit RED-tris-nitriloacetic acid (NanoTemper Technologies #MO-L008) according to manufacturer instructions. A series of 16 1:1 dilutions of unlabeled ezrin_482-586_ was prepared in the labeling buffer, producing final ezrin_482-586_ concentrations ranging from 412 μM to 12.6 nM. Two hundred nanomolars fluorescently labeled GST-His_6x_ or GST-His_6x_-CLIC5A were added to each ezrin_482-586_ dilution to achieve a final volume of 20 μl. The final concentration of the fluorescently labeled protein was 100 nM. Samples were loaded into pre-treated capillaries (NanoTemper Technologies #MO-K022) and three independent experiments were performed to quantify the dissociation constants. MST measurements were performed at ambient temperature using 40% MST power and 30% LED power with 5 s and 20 s of laser-off/on time, respectively. The K_d_ were calculated using baseline-corrected fluorescence values. Data analysis was performed using the NanoTemper MO.Affinity Analysis software (ver. 2.3; https://nanotempertech.com/monolith).

### Western blot analysis and quantification

Protein samples were denatured in Laemmli buffer containing 2.5% β-mercaptoethanol, separated by SDS-PAGE and transferred to polyvinylidene fluoride membranes. Membranes were blocked and incubated with appropriate primary and horseradish peroxidase-conjugated secondary antibodies, and protein bands were imaged with enhanced chemiluminescence (GE Amersham, Baie d’Urfe). The membranes were exposed to X-ray film (FujiMedical X-Ray Film Super Rx, Fujifilm) or imaged by iBright 750 (Invitrogen). Band densities were determined by ImageJ (https://imagej.net/ij/download.html). Original data for Western blot (WB) are shown in the supporting information section.

### Surface protein biotinylation

The Pierce cell Surface Protein Isolation Kit (#A44390 Thermo Fisher Scientific) was used according to the manufacturer’s instructions with minor modifications. Forty-eight hours after transfection, confluent COS-7 cells adherent on 100 mm culture plates were washed twice with ice-cold PBS and incubated with 10 ml of 272 μM sulfo-NHS-SS-biotin in PBS by gentle orbital agitation at 4 °C for 30 min. Biotinylation was quenched, the cells were scraped into 10 ml PBS and centrifuged at 500*g* for 3 min at 4 °C. The pellets were washed twice with PBS and lysed in 500 μl of lysis buffer (proprietary) for 30 min on ice with intermittent vortexing. The cellular debris was sedimented at 10,000*g* for 2 min in 4 °C. The resulting supernatants were applied to NeutrAvidin columns, equilibrated at room temperature for 60 min, and then spun at 1000*g* for 1 min. The resulting flow-through was kept as a fraction. The columns were then washed four times with wash buffer (proprietary) containing 300 mM NaCl and 1× protease inhibitor cocktail, followed by incubation with 400 μl 2× Laemmli buffer containing 50 mM DTT for 60 min. Eluted proteins were separated from the beads by sedimentation at 1000*g* for 1 min and subjected to WB analysis.

### Protease protection assay

Confluent COS-7 cells were washed, scraped from the plates, and pelleted (350*g* for 5 min at 4 °C) in ice-cold PBS. The pellets were resuspended in PBS with various concentrations of digitonin and trypsin-EDTA (0.02 mg/ml) in a total volume of 200 μl, followed by incubation on ice for 30 min. Controls were treated identically except for the addition of trypsin. Trypsin activity was stopped using 1× protease inhibitor cocktail, followed by incubation for additional 10 min on ice. The cells were then sedimented at 1500*g* for 10 min at 4 °C and solubilized in 2× Laemmli buffer for WB analysis.

### Differential detergent fractionation

A sample of total cell lysate was reserved from washed COS-7 cells. Washed and pelleted COS-7 cells were resuspended in cytoplasmic extraction buffer (200 μg/ml digitonin for COS-7 cells, in 40 mM Pipes, 1.2 M sucrose, 400 mM NaCl, 12.5 mM MgSO_4_, 5 mM EDTA, 1× protease inhibitor cocktail, 50 nM Calyculin A, pH 6.8), incubated by rotary shaking for 10 min (COS-7 cells) at 4 °C and then centrifuged at 2000*g* for 10 min at 4 °C. The supernatants were designated as the digitonin soluble cytoplasmic fraction. The pellets were washed once with ice-cold PBS, centrifugated at 6000*g* for 10 min at 4 °C and resuspended in membrane extraction buffer (0.5% *v*/*v* Triton X-100, 50 mM Hepes, 150 mM NaCl, 5 mM EDTA, 1× protease inhibitor cocktail, 50 nM calyculin A, pH 7.4), incubated for 30 min on ice with intermittent vortexing, followed by centrifugation at 6000*g* for 10 min at 4 °C. The resulting supernatants were designated as the Triton X-100 soluble membrane fraction, and the pellets were designated as cytoskeletal/nuclear fraction. The volume of incubation of extraction buffers was 400 μl. For WB analysis, each fraction represents equivalent starting material.

### Isolation of mouse glomeruli

All animal work was performed according to the guidelines developed by the University of Alberta Animal Care and Use Committee, and the experiments in this manuscript were approved by the committee (protocol AUP00000222). Mouse glomeruli were isolated from 4 to 6 months old WT C57/BL6 mice using differential sieving method (55), with slight modifications. Mouse kidney cortex was finely minced, suspended in RPMI 1640 medium containing 1.0 mg/ml collagenase IV (Worthington) and digested at 37 °C for 1.0 h. The digested tissue was passed through stacked 100 μm, 70 μm, and 40 μm cell strainers (BD Falcon). Contaminating tubules were removed by adhesion to cell culture plastic. Glomerular preparations were examined for purity by light microscopy and were used only if they were free of tubules (>99% pure). The glomeruli were sedimented by centrifugation at 4 °C at 4000*g* for 5 min and washed twice with cold PBS.

### Immunofluorescence and confocal microscopy

COS-7 cells were transfected with FLAG-CLIC5A cDNA and then plated on glass coverslips. Forty-eight hours later, the cells were washed with ice-cold PBS and fixed with 1% paraformaldehyde (Life Technologies) at room temperature for 15 min, followed by two washes with ice-cold PBS. The cells were then kept intact or permeabilized with 0.05% *v*/*v* Triton X-100 in PBS for 10 min on ice. The cells were then blocked with 5% *w*/*v* bovine serum albumin (BSA) in PBS for 1 h at room temperature and incubated with rabbit anti-*N*-cadherin (1:200), rabbit anti-GAPDH (1:100), or mouse anti-FLAG M2 antibodies (1:200) overnight in 4 °C. After 24 h, the cells were washed six times with PBS containing 1% *w*/*v* BSA, followed by incubation with Alexa Fluor 594–conjugated donkey anti-rabbit (1:400), or goat anti-mouse (1:400) antibodies for 45 min in the dark at room temperature. The cells were then washed four times with PBS containing 1% *w*/*v* BSA and mounted using ProLong Diamond (Thermo Fisher Scientific). Images were acquired using the Zeiss LSM710 laser-scanning confocal microscope and processed using ZEN (v 2.3; https://www.zeiss.com/microscopy/en/products/software/zeiss-zen.html).

For live-cell imaging, HeLa cells grown on 25 mm coverslips were placed into imaging chambers (37 °C, 5% CO_2_) and imaged with an Olympus IX-81 spinning disk confocal fluorescence microscope. Image acquisition was done with Volocity software (Improvision). Quantification of the plasma membrane: cytosol ratio of GFP-CLIC5A was performed as previously described (25). Pixel intensity line plots were obtained for individual cells using split red and green channels. Pearson coefficients were determined on complete images containing each containing multiple cells, using the jars colocalization finder plug-in for ImageJ.

### Rac1-GTP pull down and quantification

To precipitate Rac1-GTP, the PAK-PBD pull-down assay from Cytoskeleton, Inc. (# BK035) was used according to the manufacturer’s instructions, using cell lysates harvested from 100 mm plates and 40 mg of PAK-PBD beads. For quantification of Rac1-GTP, the G-LISA assay (# BK128, Cytoskeleton, Inc.) was used. The protein concentration was determined by Bradford assay, and 20 μg of cell lysate protein was used per data point. To deplete cell lysates of Rac-GTP, they were incubated at room temperature overnight, and the Rac-GTP depleted lysates were used to define the background and to establish the standard curve consisting of 0, 1, 3, and 6 ng constitutively active Rac1.

### YTH library screening and mapping

A Y2H protein domain screen was performed by Hybrigenics (Evry) using CLIC5A as bait and an adult mouse kidney library (cDNAs representing ∼80,000 distinct protein domains) as prey. To confirm and map direct CLIC5A interacting protein domains, the GAL4-based Y2H (Takara Bio Inc) assay kit was used according to the manufacturer’s instructions. Full-length CLIC5A (aa 1–251) and its N-terminal (aa 22–251) and C-terminal (aa 1–232) deletion mutants in the bait vector pGBKT7 were transformed into the Y2HGold yeast strain and allowed to grow for 3 days at 30 °C on synthetically defined agar medium (SD) without tryptophan (Trp^−^). Constructs encoding full-length ezrin, ezrin fragments as well as radixin, and moesin fragments in the prey vector pGAD-T7 were transformed into the Y187 yeast strain and allowed to grow for 3 days at 30 °C on SD agar medium lacking leucine (Leu−). The transformed bait and prey vector-containing yeast colonies were then mated to generate diploids and spread on Leu^−^/Trp^−^ (double dropout, DDO) agar medium, Leu^−^/Trp^−^ agar medium containing X-α-Gal (DDO/X), or Leu^−^/Trp^−^ agar medium containing X-α-Gal and the antibiotic Aureobasidin A (DDO/X/A). They were incubated for ∼3 to 5 days at 30 °C. Yeast transformed with pGBKT-7/p53 (bait) and pGAD-T7/SV40 large T antigen (prey) served as positive control, and yeast transformed with pGBKT-7/Lamin (bait) and pGAD-T7/SV40 large T antigen (prey) served as negative control. Nontransformed diploid Y2HGold/Y187 yeast served as an additional negative control.

### GST pull-down assay

The GST pull-down assay was performed in the cold room as previously reported (7, 25) with minor modifications. Briefly, COS-7 cells were scraped from the plates and evenly suspended in lysis buffer (1% Triton X-100, 20 mM Hepes pH 7.4, 0.6 M KCl, 1 mM EDTA, with 1× proteinase inhibitor cocktail [Roche] and PhosStop [Roche]), incubated for 20 min at 4 °C with end over end rotation, followed by centrifugation for 30 min at 14,000*g*. Fifty microliters of the supernatant were removed (cell lysate input) and the remaining 500 μl supernatant was precleared with glutathione beads and 12 μg GST and then incubated with equimolar concentrations of GST or GST-CLIC5A and glutathione beads on a rotary shaker for 2 h. The beads were washed twice with PBS/0.1% Tween-20 and twice with PBS. Bound proteins were eluted in 2× Laemmli buffer, followed by WB.

### Coimmunoprecipitation

Coimmunoprecipitation was performed as previously described (56) with minor modifications. Briefly, COS-7 cells were washed twice with ice-cold PBS and placed into cold immunoprecipitation lysis buffer (50 mM Tris–HCl, pH = 7.4, 150 mM NaCl, 1.0% NP-40, 0.5% sodium deoxycholate, 30 mM sodium fluoride, 40 mM β-glycerophosphate, 20 mM sodium pyrophosphate, 1 mM sodium orthovanadate, 100 nM calyculin-A, complete protease inhibitors [Roche], and PhosStop [Roche]). Cells were homogenized on ice, and centrifuged at 14,000*g* or 15 min at 4 °C. For preclearing, the supernatants were incubated for 30 min with 30 μl protein G plus/protein A-agarose beads (Calbiochem) at 4 °C, and centrifuged 12,000*g*, for 3 min. The supernatants were then incubated with goat anti-GFP antibody (1 μg/500 μl) on a rotary shaker for 2 h, followed by addition of protein G plus/protein A-agarose beads (60 μl/500 μl) from a 1:1 slurry, and overnight incubation at 4 °C on a rotary shaker. The beads were then sedimented at 1000*g* for 5 min and extensively washed as previously described (56). The sedimented beads were resuspended in 60 μl 2× Laemmli buffer and boiled for 10 min. The eluted proteins were separated by SDS-PAGE and subjected to WB analysis.

### Statistical analysis

All experiments were performed three or more separate times (biological replicates) and are reported as mean ± SD. In some experiments, technical replicates (individual data points from within a single experiment) are also reported. For experiments with more than two groups, one-way ANOVA was used followed by Dunnett’s *post hoc* evaluation for individual differences. For experiments involving two independent variables, two-way ANOVA was used followed by Tukey’s *post hoc* evaluation of individual differences. For experiments with two groups, Student’s *t* test was used. Statistical significance was defined as *p* < 0.05.

## Data availability

In addition to data reported here, replicates and original Western blots are accessible at https://doi.org/10.5683/SP3/TFEJ8S.

## Supporting information

The article contains supporting information.

## Conflict of interest

The authors declare that they have no conflicts of interest with the contents of this article.
