# Supplementary material for: CLIC5A binds to and stabilizes the open and active conformation of ezrin
Source: J Biol Chem. 2025 Aug 28;301(10):110646. doi: 10.1016/j.jbc.2025.110646 (PMC12494564; doi:10.1016/j.jbc.2025.110646)
Supplement: Supplementary Material [file mmc1.pdf]

Supporting Data
Figure S1

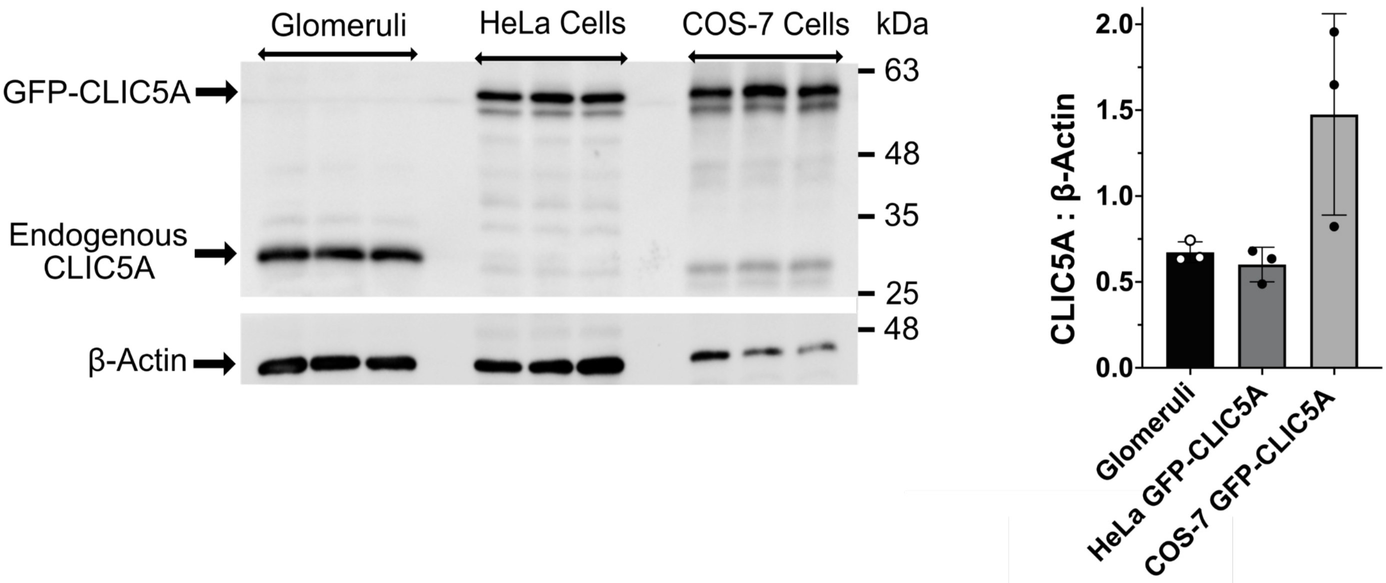

Figure S1. Similar level of endogenous CLIC5A and expressed GFP-CLIC5A . Glomeruli were isolated from 3 separate mice and adjustedto 5000 glomeruli/100 µl. 10 µl of lysate was loaded. Each lane represents a different mouse. HeLa and COS-7 cells were transfected with 2 µg cDNA/ ~ 10^6 cells. Each lane represents a distinct experiment.

Figure S2

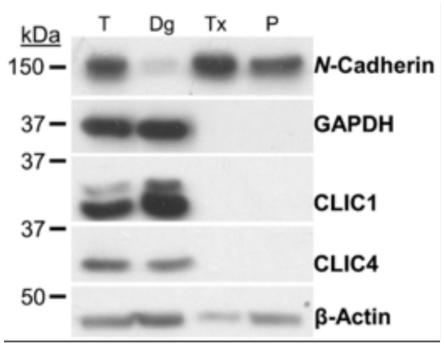

Figure S2 Like expressed CLIC5A, endogenous CLIC1 and CLIC4 are soluble proteins.
Un-transfected COS-7 cells were sequentially extracted with digitonin (Dg) and Triton X-100 followed by sedimentation of insoluble material (see Methods).
N-cadherin and GAPDH served as controls for integral membrane and soluble proteins, respectively.

Table S-1: Source of Primary Antibodies

Primary Antibodies	Host	Catalog #	Source	WB	IF	IP
N-cadherin	Rabbit	4061S	Cell Signaling, Danvers, MA, USA	1:2,000	1:200	
GAPDH	Rabbit	2118S	Cell Signaling, Danvers, MA, USA	1:5,000	1:100	
CLIC5A	Rabbit	ARP35263	Aviva System Biology, San Diego, CA, USA	1:4,000		
β-Actin	Mouse	A2228	Sigma-Aldrich, Oakville, ON, Canada	1:10,000		
FLAG	Mouse	F1804	Sigma-Aldrich, Oakville, ON	1:4,000		
Ezrin	Rabbit	3145S	Cell Signaling, Danvers, MA, USA	1:4,000		1:200
Ezrin (C-terminal)	Rabbit	Ab40839	Abcam Inc. Waltham, MA, USA	1:4,000		
Radixin	Rabbit	MA5-14886	ThermoFisher Sci. Waltham, MA, USA	1:1,000		
Moesin	Rabbit	3150S	Cell Signaling, Danvers, MA, USA	1:1,000		
pERM	Rabbit	Ab76247	Abcam Inc. Waltham, MA, USA	1:4,000		
Rac1	Mouse	ARC03-S	Cytoskeleton Inc, Denver, MA, USA	1:1,000		
HA	Rabbit	3724S	Cell Signaling, Danvers, MA, USA	1:1,000		
GFP	Rabbit	N/A	Dr. Luc Berthiaume, University of Alberta	1:4,000		
GFP	Goat	N/A	Dr. Luc Berthiaume, University of Alberta			1 μg/500 μL Lysate
NHERF2	Rabbit	9568S	Cell Signaling, Danvers, MA, USA	1:2,000		
Nephrin	Goat	AF3159	R&D Systems Minneapolis, MN, USA	1:5,000		
Rho-GDIα	Mouse	Sc-373724	Santa Cruz Biotech. Dallas TX USA	1:2000		

Table S-2: Source of Secondary Antibodies

Secondary antibodies/reagents	Conjugate	Catalog #	Source	WB	IF
Streptavidin	HRP	3999S	Cell Signaling, Danvers, MA, USA		
Goat anti-mouse IgG(H+L)	HRP	115-035-003	Jackson ImmunoResearch Lab Inc. West Grove, PA, USA	1:10,000-1:50,000	
Goat anti-Rabbit IgG(H+L)	HRP	32460	ThermoFisher Sci. Waltham, MA, USA	1:5,000-1:10,000	
Donkey anti-goat IgG(H+L)	HRP	705-035-003	Jackson ImmunoResearch Lab Inc. West Grove, PA, USA	1:5,000-1:10,000	
Rabbit anti-mouse IgG (H+L)	Alexa Fluor 594	A11032	ThermoFisher Sci. Waltham, MA, USA		1:500-1:1,000
Donkey anti-rabbit IgG (H+L)	Alexa Fluor 594	A21207	ThermoFisher Sci. Waltham, MA, USA		1:500-1:1,000
